# Effects of Intercritical Annealing Temperature on Mechanical Properties of Fe-7.9Mn-0.14Si-0.05Al-0.07C Steel

**DOI:** 10.3390/ma7127891

**Published:** 2014-12-09

**Authors:** Xianming Zhao, Yongfeng Shen, Lina Qiu, Yandong Liu, Xin Sun, Liang Zuo

**Affiliations:** 1 The State Key Lab of Rolling & Automation, Northeastern University, Shenyang 110004, China; E-Mail: zhaoxm@ral.neu.edu.cn; 2 Key Laboratory for Anisotropy and Texture of Materials (MOE), Northeastern University, Shenyang 110004, China; E-Mails: nali@smm.neu.edu.cn (L.Q.); ydliu@mail.neu.edu.cn (Y.L.); lzuo@mail.neu.edu.cn (L.Z.); 3 Pacific Northwest National Laboratory, PO Box 999, Richland, WA 99352, USA; E-Mail: Xin.Sun@pnnl.gov

**Keywords:** medium Mn steel, TRIP effect, austenite, strength, ductility

## Abstract

A medium Mn steel has been designed to achieve an excellent combination of strength and ductility based on the TRIP (Transformation Induced Plasticity) concept for automotive applications. Following six passes of hot rolling at 850 °C, the Fe-7.9Mn-0.14Si-0.05Al-0.07C (wt.%) steel was warm-rolled at 630 °C for seven passes and subsequently air cooled to room temperature. The sample was subsequently intercritically annealed at various temperatures for 30 min to promote the reverse transformation of martensite into austenite. The obtained results show that the highest volume fraction of austenite is 39% for the sample annealed at 600 °C. This specimen exhibits a yield stress of 910 MPa and a high ultimate tensile stress of 1600 MPa, with an elongation-to-failure of 0.29 at a strain rate of 1 × 10^−3^/s. The enhanced work-hardening ability of the investigated steel is closely related to martensitic transformation and the interaction of dislocations. Especially, the alternate arrangement of acicular ferrite (soft phase) and ultrafine austenite lamellae (50–200 nm, strong and ductile phase) is the key factor contributing to the excellent combination of strength and ductility. On the other hand, the as-warm-rolled sample also exhibits the excellent combination of strength and ductility, with elongation-to-failure much higher than those annealed at temperatures above 630 °C.

## 1. Introduction

With the rapid increase in the number of motor vehicles worldwide and the associated environmental impact, automobile lightweighting has become an urgent global initiative. As a candidate material class to reduce weight without sacrificing passenger safety, TRIP (Transformation-induced-plasticity)-aided steels have received much attention in recent years due to their outstanding combination of strength and ductility. The excellent performances of this class of steels are closely related to both the multiphase character of their microstructure and the TRIP effect, *i.e.*, the martensitic transformation of the austenite induced by deformation [1]. Good ductility results from ductile and soft ferrite and retained austenite (RA), while high strength originates from the martensite induced by martensite transformation from RA [1,2,3]. Martensite transformation leads to an increase of strain hardenability because the mechanical properties difference between retained austenite and martensite results in increasing strain from the shear and dilatation associated with transformation [4]. The TRIP effect can significantly improve the formability and energy absorption of the material. During intercritical annealing, ferrite inversely transforms into austenite phase, which converts to martensite at the stage of subsequent quenching. The amount and stability of RA at room temperature are closely associated with the intercritical annealing (IA) temperature [5,6,7]. The stability of the austenite at room temperature is mainly controlled by carbon enrichment as a result of the specific heat treatment conditions and parameters. For example, Jacques *et al.* focused their investigations on the influence of isothermal bainite holding temperature on the retained austenite volume fraction, carbon content and the overall mechanical properties of the TRIP-assisted steels [8,9]. In order to obtain a microstructure with a specific amount and stability of RA, quenching and partitioning (Q&P) treatments [3,8] have proven effective in producing high-strength steels with a mixed microstructure of tempered martensite and retained austenite [3,8]. The typical Q&P process consists of a first quench (from either full or partial austenization) to a temperature between the martensite start and martensite finish temperature, and a subsequent isothermal treatment at the same or higher temperature to promote the partitioning of carbon from the supersaturated martensite to the retained austenite.

On the other hand, excellent combinations of strength and ductility have been extensively achieved in metals and alloys by engineered microstructure features, including bimodal grain size distribution [10], nanoscale twin [11,12,13], and dispersed distribution of nanoparticles [14]. Most of the existing investigations on TRIP steels mainly focus on improving the mechanical properties by adjusting the volume fraction ratio of ferrite to austenite, which can be achieved by controlling chemical compositions and/or heat treatment processes [15,16,17,18]. Only a handful of works have noted that excellent mechanical properties of TRIP steels can be obtained by controlling the morphology of austenite [19,20]. In the current work, we demonstrate that excellent combination of strength and ductility of a medium Mn TRIP steel can be achieved by controlling the volume fraction and the morphology of austenite through hot rolling followed with warm rolling (without the annealing process), replacing the traditional three-stage processes including hot rolling, cold rolling, and annealing.

## 2. Experimental Methods

### 2.1. Sample Preparation

**Figure 1:**
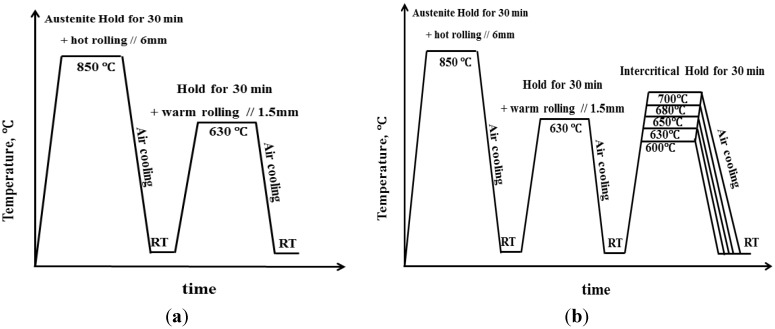
Schematic illustration of steps in rolling and heat treatment processes of medium-manganese steels in present work (**a**) for the warm-rolled steel; and (**b**) for the warm rolled steels heat-treated with intercritical annealing.

The alloy was melted in a vacuum induction furnace, and an ingot (300 × 200 × 20 mm^3^) was cast. The alloy’s chemical composition, measured by inductively coupled plasma mass spectroscopy, is listed in Table 1. A plate was cut from the ingot, homogenized in an air furnace at 1250 °C for 1 h to remove any inhomogeneous microstructures evolved during solidification. Subsequently, the plate was heated to 900 °C and held at that temperature for 30 min for full austenization. To obtain a warm-rolled steel, hot rolling was conducted at 850 °C in six passes with a reduction of ~12% each pass to a thickness of 6 mm (ε = 70%). After each pass, necessary reheating was performed to ensure the plate soaked at 850 ± 5 °C for 5min before the next pass. After six passes, the plate was cooled to room temperature in air. The obtained plate was then heated up and kept at 630 °C for 10 min before it was warm-rolled to a thickness of 1.5 mm in seven passes (ε = 75%). Again, after each pass, necessary reheating was performed to ensure the plate soaked at 630 ± 3 °C for 5 min before the next pass. After seven passes, the plate was cooled to room temperature in air (Figure 1a). Next, to investigate the effect of intercritical annealing, the plate was divided into five equal parts and annealed at 600 °C, 630 °C, 650 °C, 680 °C, 700 °C for 30 min with a continuous annealing machine (CAS30011, General Furnace Manufacturing Co., Ltd., Shenyang, China), followed by air-cooling to room temperature (Figure 1b).

**Table 1:** Chemical composition of the investigated steel (wt.%).

C	Mn	Si	Al	S	P	Fe
0.07	7.9	0.14	0.05	0.002	0.003	Bal

In this study, warm rolling was conducted at a relatively high temperature to make the rolling process more easily operable by inhibiting crack/void initiation which is prone in cold rolling. In addition, the reduction of rolling forces with the increase in rolling temperature is also beneficial in prolonging the service life of rollers.

To determine the start point of martensitic transformation, cylindrical dilatometric samples with 3 mm-diameter and 10 mm-length were tested with a push-rod L78-RITA dilatometer (Linseis Messgeraete, Selb, Germany). The sample’s temperature is measured by a thermocouple welded to its surface using a precision welder and the jig supplied by the dilatometer manufacturer. The heating and austenitization treatments were carried out under a vacuum of 5 × 10^−2^ Pa, and the cooling was achieved using argon gas.

### 2.2. Mechanical Property Characterization

To examine the effect of heat treatment on the mechanical properties of the steel, the as-treated steel sheets were cut into the dog-bone shaped specimens with a gauge length of 15 mm and a width of 4 mm, and the final sheet thickness is 1.2 mm after polishing. Uniaxial tensile tests were performed on an AG-X plus mechanical testing system at a constant strain rate of 1 × 10^−3^·s^−1^ under room temperature. A calibrated contactless MTS LX300 laser extensometer (MTS Systems Corporation, Eden Prairie, MN, USA) was used to measure the sample strain upon loading. Note that the dimensions of the tensile specimens in this study do not follow the American Society for testing and materials (ASTM) standard, and the higher length to width ratio may result in slightly overestimated properties in comparison with those reported by other authors [18,21].

Micro-hardness of each phase was determined using a Triboindenter (Hysitron Incorporated, Minneapolis, MN, USA) equipped with a diamond Berkovich tip with a pyramidal tip shape and a tip radius of approximately 100 nm. The specimens were loaded to the maximum load of 2 mN and with a loading rate of 0.4 mN/s and held constant for 10 s. The average value for each phase was calculated from 12 indents.

### 2.3. Microstructure Characterization

Specimens were cut from the heat-treated sheet and the gauge of the deformed samples for X-ray diffraction (XRD) analysis in determining RA volume fractions. The specimens were ground gradually using silicon papers with different grades, chemically cleaned using a mixture of HCl and HNO_3_ in 2:1 ratio, further swabbing by few drops of ethanol to improve precision of analysis. The measurement of RA volume was performed on X’Pert PRO diffractometer (PW3040/60, Panalytical B.V., Almelo, The Netherlands) with a sample stage for Co anode (wavelength *k* = 0.1789 nm) and X-ray detector, operating at 40 kV and 40 mA. The penetration depth of X-rays (Co radiation) into steel is a few micrometers. Samples were scanned at a step size of 0.02 deg per second and a 2θ ranging from 40 to 100 deg. The volume fraction of retained austenite phase was determined with the integrated intensity of (111)γ, (200)γ and (220)γ, and ferrite peaks (110)α and (200)α with the following Equation [22]:

(1) $$V_{\text{γ}}=1.4I_{\text{γ}}/(I_{\text{α}}+1.4I_{\text{γ}})$$

Here *V*_γ_ is the volume fraction of RA; *I*_γ_ and *I*_α_ is the integrated intensity of peaks of RA phase and ferrite phase, respectively.

The microstructure was characterized using scanning electron microscope (SEM) (FEI Quanta 600, PHILIPS, Hillsboro, OR, USA) operated at a voltage of 20 kV. The last specimen-preparation step was electrolytic polishing with an electrolyte consisting of 15% perchloric acid, 5% distilled water, and 80% ethanol (in v/v) at 20 V for 25 s. The electron-back-scattered-diffraction (EBSD) analysis was conducted with an acceleration voltage of 20 kV, sample tilt angle of 70°, and working distance of 7 mm. To obtain a representative value for the phase fraction, it is important that the EBSD data covers a large area and includes enough points. An area of 1.0 × 1.0 mm^2^ was covered per specimen. Maps were measured on a hexagonal grid with step sizes of 0.1 µm. The EBSD data was collected on a Ultra-55 field-emission SEM (Zeiss Ltd., Braunschweig, Germany) and evaluated by the orientation imaging microscopy (OIM) software (Oxford Instruments PLC., Oxfordshire, UK).

Transmission electron microscopy (TEM) techniques (Tecnai G^2^ 20, FEI Company, Hillsboro, OR, USA) were used to investigate the micro-structural morphologies associated with the various heat treatment processes and deformation state for the studied steel. Specimens for TEM observation were cut from the annealed samples and the tips of deformed samples close to the fracture surface, mechanically polished from both sides to a final thickness of about 50 µm. Subsequently the samples were polished with diamond paste, and then the foils were thinned using a double jet electrolytic polisher at a voltage of 50 V and a temperature below −10 °C. The electrolyte consists of 10% perchloric acid and 90% (v/v) ethanol. TEM observations were performed in a field-emission-gun (FEG) Tecnai G^2^ 20 microscope operating at an accelerated voltage of 200 kV.

## 3. Results

Ac_1_ and Ac_3_ temperatures of the steel are determined by the dilatometer as 585 °C and 720 °C, respectively. The start point of martensitic transformation occurs at 300 °C for a range of cooling rates of 0.1–60 °C/s.

**Figure 2:**
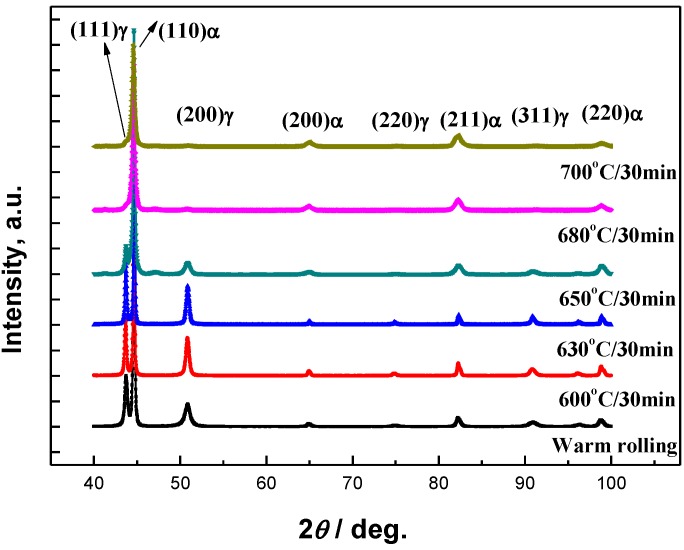
XRD patterns for the warm-rolled steel along with those after various heat treatment processes.

Figure 2 is the XRD patterns of the investigated steels as warm-rolled state along with those annealed under various temperatures for 30 min. The austenite volume fraction is calculated with Equation (1) as 35%, 39%, 36%, 19%, 9% and 7% for the steels as warm-rolled and as annealed at 600 °C, 630 °C, 650 °C, 680 °C, 700 °C for 30 min, respectively. In the range studied, the RA volume fraction decreases with increasing annealing temperature. This is similar to the measured austenite volume fraction reported by Lee and De Cooman [5] for a 6Mn steel, and the reason may lie in the variations of C and Mn concentration in austenite, affecting austenite’s thermal stability.

**Figure 3:**
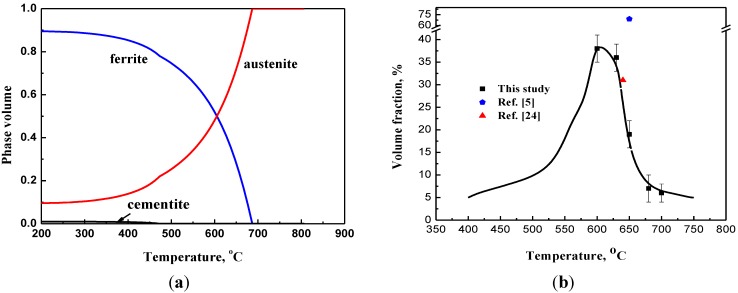
(**a**) Phase fractions were predicted by Thermal-Calc™ using JMatPro database; (**b**) Final austenite fraction as a function of annealing temperature for a Fe-7.9Mn-0.14Si-0.05Al-0.07C Steel (solid line, predicted, black square, measured data).

The phase fractions predicted by Thermal-Calc™ using JMatPro database for this steel are shown in Figure 3a. One can clearly see that the highest austenite fraction is 52% at temperature of 630 °C, balanced with ferrite. Below 450 °C, some cementite (<1%) can been observed. However, the austenite fraction decreased with decreasing temperature and the remaining fraction was stabilized by Mn and C. It has been reported that the diffusion distance of Mn is 280 nm in bcc iron at 640 °C [6], at which the greatest RA fraction of 31% was obtained for a 0.11C-5.7Mn steel. Meanwhile, a maximum room temperature austenite volume fraction of 69% was obtained at an intercritical annealing temperature of 660 °C in the medium Mn TRIP steel with a nominal chemical composition of Fe-0.3C-6.0Mn (wt%) [5]. In our study, we used the Thermal-Calc™ using JMatPro database to evaluate the change of final austenite volume fraction with increasing heat treatment temperatures. According to Speer *et al.* [23], the inputs to this calculation include the carbon content of the steel, the Ms temperature, and the cooling rate [16], assumed that only carbon can reach uniform chemical potential (*i.e.*, constrained carbon equilibrium (CCE)) at the interface between austenite and martensite whereas other atoms cannot move [23]. Surprisingly, both the predicted (dark line) and measure results (solid dots) are similar to those obtained from quenching processes, with the highest volume fraction of 39% retained austenite predicted for the sample annealed at 600 °C for 30 min (Figure 3b).

**Figure 4:**
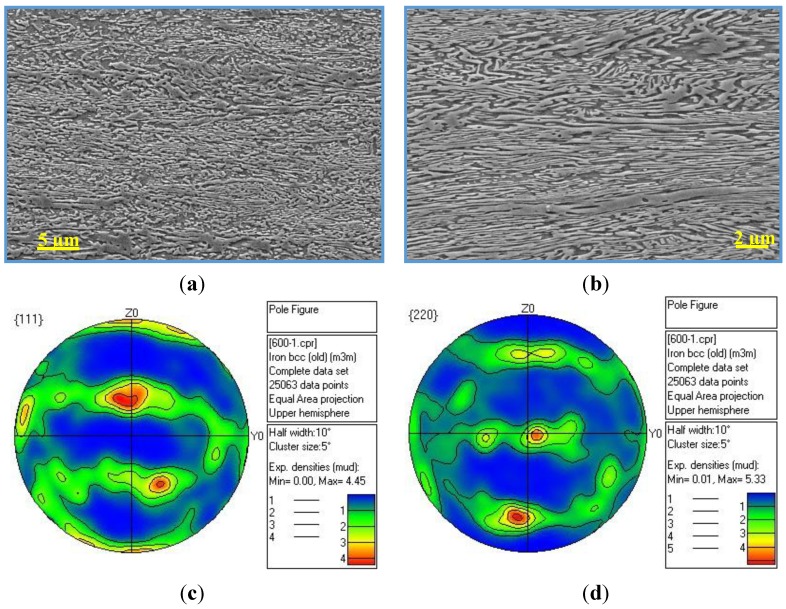
(**a**) Low-; and (**b**) high-magnification SEM micrographs show the morphology and arrangement of ferrite (light grey) and austenite (dark grey), in the warm-rolled plate after annealing at 600 °C for 30 min (**c**); and (**d**) are the corresponding (111) and (220) pole figures obtained using EBSD analysis.

Though the numerous heating and cooling cycles during warm-rolling can strongly affect the microstructures of the warm-rolled steel, postmortem SEM observations proved that the microstructures consist of mainly ferrite and austenite, accompanied with some martensitic packets. Hence, subsequent intercritical annealing was conducted to promote the transformation of martensite into austenite. SEM and TEM observations indicated that, after intercritical annealing at 600 °C for 30 min, the warm-rolled steel mainly consists of light grey ferrite and dark grey austenite (Figure 4a,b). It is clear from Figure 4a,b that there is a texture in the warm-rolled plate after annealing at 600 °C for 30 min. Consequently, the texture measurements of were carried out in a plane perpendicular to the transverse direction of the annealed plate, as shown in Figure 4c,d. One can see that γ-fiber, {111}<112>, is the main component, with an intensity of 5.33 times random. This texture can slightly affect the calculated values of RA by using Equation (1), resulting in an overestimated RA volume fraction in this study.

**Figure 5:**
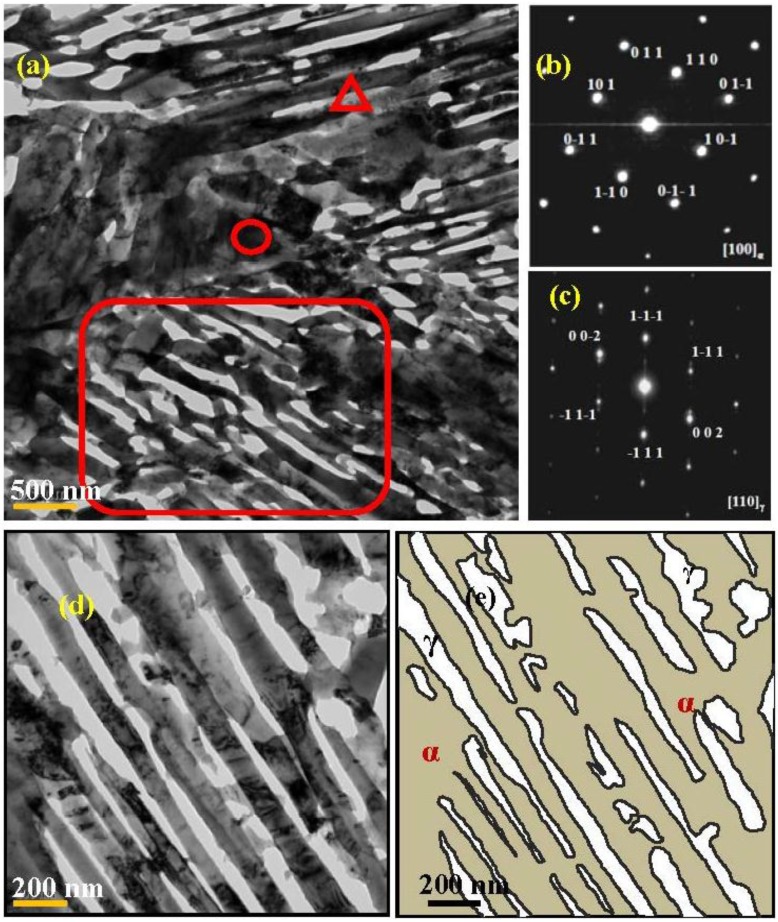
(**a**) TEM micrograph, and selected-area-electron-diffraction (SAED) patterns for the circle; (**b**) and triangle; (**c**) indicated areas in the warm-rolled plate after annealing at 600 °C for 30 min; (**d**) shows the close observation to the square indicated area in (**a**); (**e**) A schematic illustration of (**d**) to help differentiate and measure the thicknesses of ferrite and austenite. α: ferrite; γ: austenite.

TEM observations reveal that both phases exhibit ultra-fine lamellar microstructures with thickness varying from tens to hundreds of nanometers (Figure 5). The average lamellae thickness is about 350 nm. In the selected-area-electron-diffraction (SAED) shown in Figure 5b,c, the dark and bright area were discerned as ferrite and austenite, respectively. Interestingly, one can clearly see two phases alternatively arranged and the majority of austenite have been eroded away (Figure 5a,d). The key reason can be attributed to the high Mn and C concentrations in austenite. During the electrolytic polishing, the different potential between the ferrite and RA would result in a difference in the erosion sequence.

**Figure 6:**
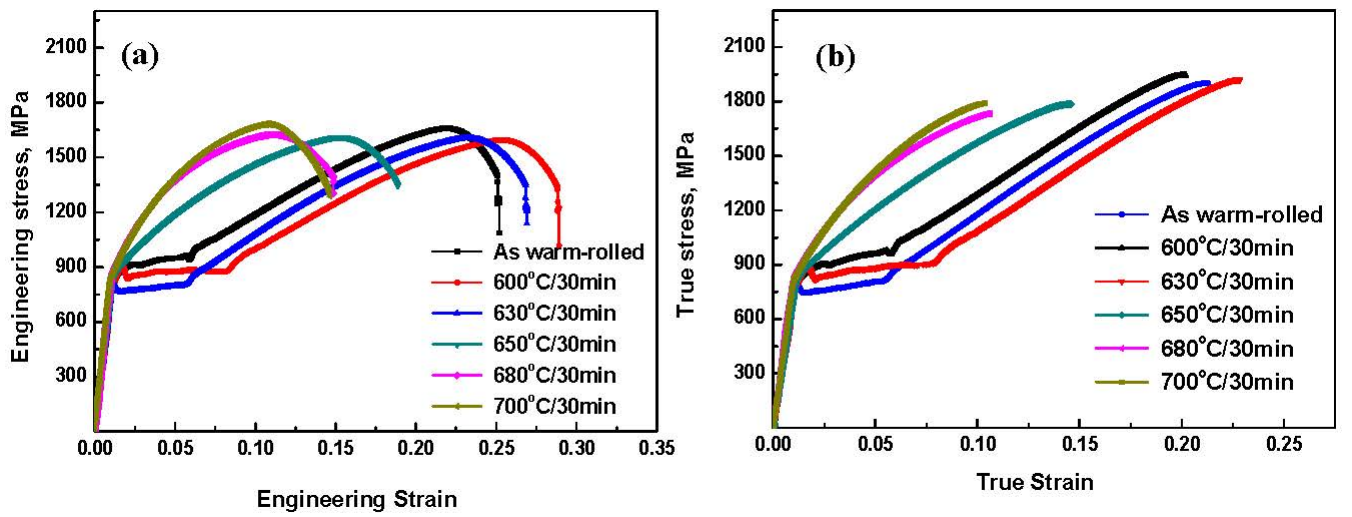
The engineering stress—strain (**a**) and true stress—strain curves (**b**) for the samples obtained by various heat treatment processes.

Tensile testing results reveal that the annealing temperature dramatically influences the mechanical properties of the studied steels. All engineering stress-strain and true stress-strain curves for the investigated samples are presented in Figure 6. Several significant observations can be made from Figure 6. First, the as warm-rolled steel exhibits an ultimate tensile strength (σ*_UTS_*) of 1670 MPa and a moderate elongation-to-failure (ε*_f_*) of 0.24. The samples show obviously different behaviors with increasing annealing temperatures. At the lowest annealing temperature of 600°C, the sample exhibits the highest ε*_f_* of 0.29 and a slightly decreased σ*_UTS_* of 1600 MPa. With increasing annealing temperature to 630 °C, the ε*_f_* decreases to 0.27 while the σ*_UTS_* remains the same. Finally, when the annealing temperatures go beyond 630 °C, the ε*_f_* significantly decreases to ≤ 0.18 due to the increasing amount of martensite and reduced amount of RA in the samples. Notice that the σ*_UTS_* is almost the same for all the samples. From Figure 6 one can clearly see the warm-rolled sample with 600 °C annealing temperature has the best combination of ultimate tensile strength and elongation to failure. It is worthwhile pointing out that the warm-rolled specimens after annealing at the lower temperature for 30min exhibit good ductility (ε*_f_* ≥ 0.27), high yield strength and extremely high σ*_UTS_* of 1600 MPa, which is over two times higher than that reported for the martensitic 8Mn steel with the same amount of Mn [25].

## 4. Discussion

### 4.1. Heat Treatment

The warm rolling was carried out above *A*c_1_ (585 °C). It means that the austenite formed during the warm-rolling process and the part subsequently transformed into martensite upon cooling. Consequently, the intercritical annealing was performed to push the inversely transformation of the martensite into austenite. The evolution of the volume fraction of retained austenite was investigated by varying the annealing temperature, seeking a good balance of phase ratio. Unfortunately, it was proved that the intercritical annealing had played a limited role in increasing the ductility, in comparison with the as warm-rolled steel. Furthermore, it is inevitable to make it difficult to monitor forming and dissolution of carbides, due to the fact that many heating and cooling steps were applied. The reduced amount of RA with increasing annealing temperature may be due to the following reasons: an increase in intercritical temperature can facilitate the formation and growth of austenite, correspondingly leading to an increasing in austenite volume fraction at the stage of annealing. It is well established that the mechanical stability of retained austenite is proportional to its carbon concentration [20]. The amount of carbon atoms in the steel remains the same. Therefore, the concentration of C in austenite is lower for the higher annealing temperatures. Thus, the high volume fraction of austenite formed at the annealing stage should associate with a weak stability, which mainly results from a low average carbon content of austenite. Consequently, more austenite should transform to ferrite or martensite during the subsequently quenching process. It is conceivable that the RA should decrease with increasing intercritical temperature over 600 °C.

**Figure 7:**
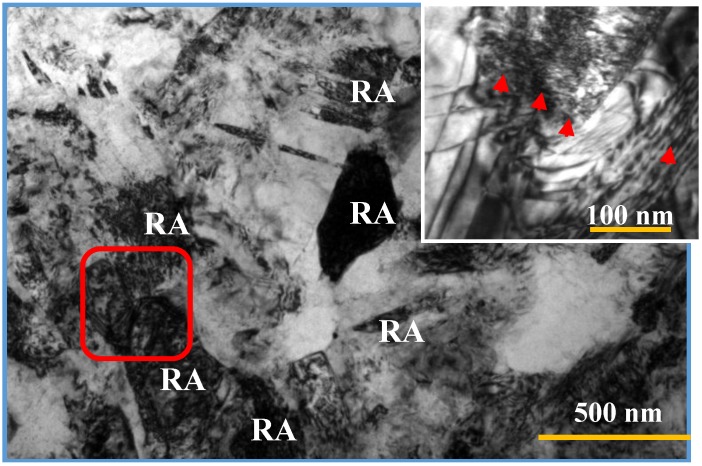
TEM micrograph of the microstructure in the warm-rolled steel after annealing at 600 °C for 30 min. The inset is a close observation to the morphology in the square area, showing the dense dislocation arrays and tangles (indicated by arrows) in the RA.

In addition, it is well recognized that the dislocations are preferential nucleation sites for random precipitates since they are preferred diffusion paths of the atoms. Dislocations are easily generated in the austenite during the warm rolling and will remain present in the RA with a high density due to the low temperature, as shown in Figure 7. The easy segregation of carbon to dislocations in the RA because they act as diffusion pipelines of atoms render a carbon content as high as 3–4 wt.% around the dislocations as reported in the literature [24]. Hence the low annealing/austempering temperature here is helpful in maintaining a high dislocation density, thereby enhancing the RA stability [26]. Overall speaking, as the annealing temperature exceeds 630 °C, a mixture of ferrite-martensite-austenite is obtained, and the RA volume fraction decreases rapidly to 7% for annealing temperature of 700 °C.

### 4.2. RA Volume Fraction

The values, predicted by Thermal-Calc™ using JMatPro database, fit well with the measured volume fractions of RA and linearly decrease with increasing annealing temperatures, providing the temperature over 600 °C. However, it should be noted that the retained austenite volume fraction is about 30% after warm rolling and cooling (at room temperature), which is dramatically higher than the value of ~5% according to the equilibrium calculations for the retained austenite at 400 °C. Therefore, these calculations should be cautiously used for the warm-rolled samples have a large fraction of austenite already before intercritical annealing. The reason might be associated with the enhance stability of austenite resulted from the warm rolling. In addition, the calculated values are only as a function of intercritical annealing temperature, whereas the measured data reveal the combination effect of not only the warm rolling but also the intercritical-annealing temperature along with the quenching processes. As discussed in the last paragraph, the RA fraction (*i.e.*, the final volume fraction of austenite) may be related to a number of factors including C and Mn concentration in austenite, and dislocation density, *etc.* The warm-rolling process should enhance the diffusion of C and Mn into the austenite. Meanwhile, the increasing dislocation density retards the inverse diffusion of C and Mn during the quenching. To obtain a high volume fraction of RA, the methods should be explored to enhance the thermal stability of RA. In this study, the low annealing temperature of 600 °C is beneficial for achieving the RA fraction as high as 39%. The reason may be closely related to diffusion kinetic of C and Mn during the transformation of austenite, resulting from different initial quenching temperature with the same duration. A further investigation should be performed to verity this declaration.

### 4.3. Mechanical Properties

**Figure 8:**
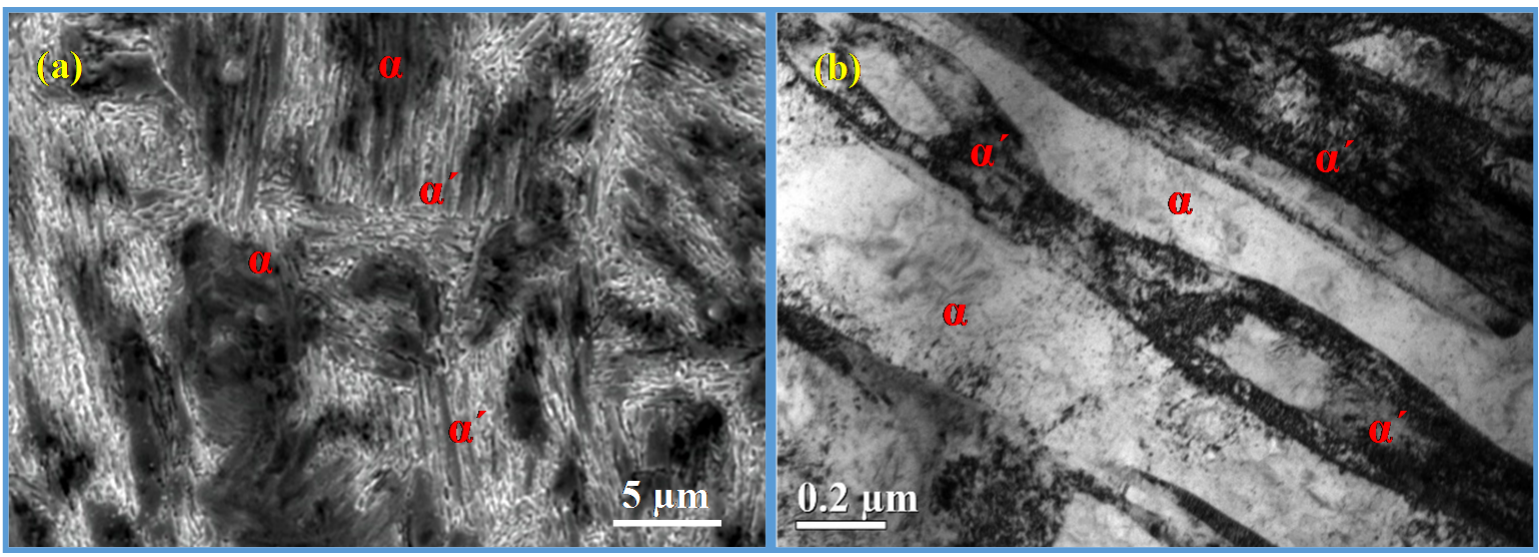
(**a**) SEM and (**b**) TEM observations of the deformed areas indicated that, after deformation, there were merely martensite and ferrite in the warm-rolled sample annealed at 600 °C for 30 min. α΄: martensite; α: ferrite.

The high strength in the studied steel can be partly attributed to the strain-induced martensitic transformation of the high volume fraction of austenite, while the other part can be related to the ultra-fine microstructure features (*i.e.*, size and morphology effects). Formation of hard martensite phase induced by deformation provides an evolving composite structure with hard martensite secondary phase in the soft ferrite matrix. Figure 8 depicts the microstructure at the end of tensile deformation, containing mainly martensite and ferrite. In TRIP steels with 100% metastable austenite, the transformation process from austenite into martensite provides a localized work-hardening effect, which delays the onset of localized necking and has been shown to be particularly effective in enhancing the uniform ductility [27]. However, the situation should be more complex in the multiphase TRIP-assisted steels. For example, various authors discussed that the strength-ductility balance at the onset of necking is mainly influenced by the rate of creation of supplementary dislocations in ferrite during the transformation of RA and the stability of RA is the key factor influencing the work-hardening behavior [28,29]. *In situ* high-energy X-ray diffraction demonstrated that the relative soft ferrite phase offers the necessary plasticity, while the hard bainite phase forms the strengthening skeleton for the optimized mechanical behavior of TRIP-assisted steel [30]. Tirumalasetty and co-authors suggested that austenite grains positioned at grain boundaries between multiple ferrite grains were apt to transform, whereas those embedded completely in larger ferrite grains underwent rotations [31], playing an equal role as the contribution of the austenite martensite transformation to contribute an additional ductility to the TRIP-assisted steels [31].

**Figure 9:**
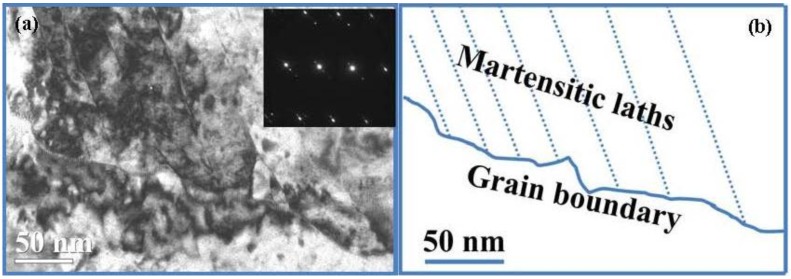
(**a**) TEM micrograph and the corresponding SAED patterns (the inset) indicating the martensite laths in the warm-rolled plate after annealing at 650 °C for 30 min; (**b**) A schematic illustration to help differentiate the martensite and ferrite.

On the other hand, yield point discontinuity (Luders strain) and the extent of yield point elongation can be observed for the as warm-rolled samples, and the annealed samples at temperatures of 600 °C and 630 °C. Interestingly, no Luders strain can be seen for the annealed samples at temperatures over 630 °C. The key reason should be attributed to the difference in the amount of soft phase, *i.e.*, the ferrite. The variations of the amount of Luders strain might depend on the volume fraction of the ferrite, which is the highest in the sample annealed at 600 °C for 30 min. Whereas the absence of Lüders bands for the higher annealing temperatures (650 °C, 680 °C, and 700 °C) is more likely to be a consequence of the presence of martensite. Though it is a challenge to distinguish the martensite from the ferrite, due their close lattice parameters and misorientation, the series of careful TEM observations proved that some martensitic laths had occurred in the warm-rolled steel after intercritical annealing at 650 °C for 30 min, as shown in Figure 9. In contrast, it is difficult to find the martensite in the warm-rolled steel after intercritical annealing at 600 °C for 30 min (Figure 5).

To elucidate the origin of good mechanical properties, nanoindentation has been performed to evaluate the hardness of each phase within the ultra-fine lamellar microstructures as shown in Figure 5. The results indicate that the hardness of ferrite and austenite is 2.25 GPa and 3.27 GPa, respectively. With Tabor’s expression [32], the hardness *H*_v_ of a material can be related to flow stress at 8% tensile strain as

(2) $$H_{V}=3{\text{σ}}_{\text{ε}=0.08}\approx 3{\text{σ}}_{y}$$

For the material with duplex constituents/phases, the average hardness can be calculated using the classical rule of mixture (ROM) [33,34]:

(3) $$H_{V}=f_{x}H_{Vx}+f_{y}H_{Vy}$$

where $f_{x}$ and $f_{\text{y}}$ are the local x- and y-constituent volume fractions in the tested regions; and $H_{Vx}$ and $H_{Vy}$ are the hardness for bulk X and Y crystals. Consequently, the yield stress of the studied steel can be estimated as follows:

(4) $${\text{σ}}_{Y}=f_{\text{α}}\frac{{H_{V}}^{\text{α}}}{3}+f_{\text{γ}}\frac{{H_{V}}^{\text{γ}}}{3}=900MPa$$

Here $f_{\text{α}}$, ${H_{V}}^{\text{α}}$ and $f_{\text{γ}}$, ${H_{V}}^{\text{γ}}$ are the volume fraction and hardness of ferrite and austenite, respectively. This value is well consistent with the tensile test of the warm-rolled sample after annealing at 600 °C for 30 min. It should be noted that this equation does not take any morphological aspects into account, but it approximates the yield strength reasonably. As far as the σ*_UTS_* is concerned, this equation should be not appropriate because the morphology of the RA can significantly affect the work-hardening behavior of the TRIP-assisted steels [19,20].

On the other hand, one may note that this specimen exhibits ultra-fine lamellar microstructures (Figure 5), and the acicular structure is believed to occur during the reverse transformation from martensite when the austenite nucleates at a lath boundary [5]. The evaluated average thickness of the lamellae is about 350 nm, incorporated with the ferrite matrix and the RA laths. According to the well-known Hall-Petch (H-P) relationship, the lower yield point of low-carbon steels can be related to grain size as:

(5) $${\text{σ}}_{Y}={\text{σ}}_{0}+k{\text{λ}}^{-0.5}$$

Here σ_Y_ and σ_0_ are the yield stress and friction stress respectively; *k* is the slope of the H-P relation; and λ is the average lamellae thickness of the ferrite matrix (~350 nm). Following on previous work [35], the value of σ_0_ and *k* is 70 MPa and 16 MPa/mm^−0.5^, respectively. Consequently, σ*_Y_* is estimated to be around 920 MPa, which is very close to that measured from tensile test. This result supports the previous assumption that it is indeed the ferrite that determines the initial yielding behavior (Luders band) of the as warm-rolled steel and those annealed at the low temperatures (600 °C, 630 °C) for 30 min.

A hardening effect arising from the transformation product as an obstacle to slip can be expressed as a function of volume fraction of martensite [36]:

(6) σ∝f⋅Δσ

Here σ is the flow stress of material; and Δσ is the strength difference between the austenite and the martensite. For the studied material, the hardness values of the austenite matrix and martensite phases are determined as 3.27 GPa and 4.7 GPa. At a true strain of 0.23 (1900 MPa), the total volume fraction of martensite is ~37%. According to Equation (6), the increment of stress is about 462 MPa resulting from the newly formed martensite, which is very close to the half of stress from strain hardening. Obviously, the enhanced work-hardening ability of this steel mainly results from the TRIP effect, *i.e.*, the steady deformation-induced transformation of austenite, which leads to a relaxation of the stress concentration and promotes the strain-hardening ability, delaying the necking and increasing the uniform elongation [36]. The good strain of the investigated steel can be attributed to the prominent feature of the warm rolled sample revealing the significant γ–fiber, which is well proved to be beneficial for improving the formability and ductility of the materials [37,38]. The present results suggest that the traditional three-stage processes including hot rolling, cold rolling and annealing can possibly be replaced with the two-stage process (hot rolling and warm rolling).

## 5. Concluding Remarks

By optimizing the heat treatment processes, excellent combination of strength and ductility has been achieved for a medium Mn steel. The steel has 39% austenite in its microstructure, and its high ductility originates from the ultra-fine lamellar austenite and acicular ferrite structure, which have been obtained by warm rolling and subsequent intercritical annealing at temperature of 600 °C. Meanwhile, the as warm-rolled steel exhibits an ultimate tensile strength (σ*_UTS_*) of 1670 MPa, high yield strength, and a comparable elongation-to-failure (ε*_f_*) of 0.24. This result is extremely inspiring in comparison with the traditional three-stage processes including the hot rolling, cold rolling, and the annealing treatments. The increasing annealing temperature results in decreased ductility due to the increasing martensite volume fraction of the obtained steels. While deformation-induced martensitic transformation of the high volume fraction austenite definitely contributes to the high strength and good ductility, contributions from grain size and morphology have also been estimated. Transformation-induced martensite provides an evolving composite structure with a hard secondary martensite phase within the soft ferrite matrix, while the ultra-fine lamellar microstructures result in a hardening effect following the classical Hall-Petch relationship.
